# The role of cigarette smoke-induced pulmonary vascular endothelial cell apoptosis in COPD

**DOI:** 10.1186/s12931-021-01630-1

**Published:** 2021-02-05

**Authors:** Qing Song, Ping Chen, Xiang-Ming Liu

**Affiliations:** Department of Respiratory and Critical Care Medicine, The Second Xiangya Hospital, Research Unit of Respiratory Disease, Diagnosis and Treatment Center of Respiratory Disease, Central South University, 139 Renmin Middle Road, Changsha, 410011 Hunan China

**Keywords:** Chronic obstructive pulmonary disease, Cigarette smoke, Epigenetic, Gene regulation, Molecular biology, Apoptosis, Endothelial cell

## Abstract

Chronic obstructive pulmonary disease (COPD) is one of the most common chronic respiratory diseases with high morbidity and mortality. It has become the fifth most burdened and the third most deadly disease in the global economy and increases year by year. The prevention and treatment of COPD are urgent. Smoking is the main and most common risk factor for COPD. Cigarette smoke (CS) contains a large number of toxic substances, can cause a series of changes in the trachea, lung tissue, pulmonary blood vessels, and promotes the occurrence and development of COPD. In recent years, the development of epigenetics and molecular biology have provided new guidance for revealing the pathogenesis, diagnosis, and treatment of diseases. The latest research indicates that pulmonary vascular endothelial cell apoptosis initiates and participates in the pathogenesis of COPD. In this review, we summarize the current research on the epigenetic mechanisms and molecular biology of CS-induced pulmonary vascular endothelial cell apoptosis in COPD, providing a new research direction for pathogenesis of COPD and a new target for the diagnosis, treatment, and prevention of COPD.

## Introduction

Chronic obstructive pulmonary disease (COPD) is a chronic respiratory disease caused by a variety of factors. It is characterized by chronic inflammation of the airways, lung tissue, and pulmonary blood vessels. Long-term inflammation causes remodelling of the airway structure and subsequent restriction in respiratory airflow. The development of restricted respiratory airflow is progressive, and the airflow restriction is irreversible, even after removing the risk factors. Eventually, it seriously affects the quality of life of patients, endangering people's health [1–4]. The latest research data show that the incidence, disability, and mortality of COPD are high, and there is a rising trend year by year. COPD has become a serious worldwide public health problem and one of the major risk factors for death in the global population. The number of patients with COPD is nearly 299.4 million adults in worldwide [5–8]. According to the Global Burden of Disease Study, 3.2 million people died due to COPD in 2017, which represented a more than 23% increase in deaths compared with 1990 [9].

Current research shows that smoking, biofuels, indoor and outdoor air pollution, and industrial dust are the major environmental risk factors for COPD [10]. Cigarette smoke (CS) contains many harmful ingredients, which have a stimulating effect on the respiratory tract. Studies have demonstrated that long-term smoking can destroy the structure of the air duct wall, damage the septum of the alveolar wall, and cause interstitial fibrosis [11–14]. In addition, smoking causes increased secretions from mucous glands and obstructive bronchiolitis, which aggravates the progression of lung tissue lesions [15, 16]. At the same time, CS can stimulate lung tissue to produce a large amount of reactive oxygen species (ROS), which can lead to an imbalance of the oxidation and antioxidant systems. This finally causes cell dysfunction and induces cell apoptosis [17–19].

Smoking is an established risk factor for COPD. The latest epidemiological data shows that smoking more than 20 packs a year triples the prevalence of COPD in China [20]. In addition to active smoking, passive smoking is also related to the occurrence of COPD [21]. Research report shows that the prevalence of patients with COPD who have never smoked is also high [20, 22, 23].

Airway inflammation, oxidative stress, and lung emphysema are the main mechanisms of the onset of COPD [24]. Recent studies have shown that pulmonary vascular endothelial cell apoptosis also initiates and participates in the pathogenesis of COPD [25]. In this review, we summarize the current research on the epigenetic mechanisms and other molecular biology of CSE/CS-induced pulmonary vascular endothelial cell apoptosis in COPD.

### COPD and pulmonary vascular endothelial cell apoptosis

Apoptosis refers to the physiological or pathological stimulating signals of the cell to the environment, such as DNA damage and oxidative stress. It’s a kind of active and orderly gene control, resulting from environmental changes or mitigation of natural death [26]. The process of apoptosis is complicated and it is a process that is strictly regulated by multiple genes and molecular signals. It involves a series of changes of molecular signal pathways. This gene-controlled apoptosis is highly conserved among different species. Common apoptotic genes include the Bcl-2 family, caspase family, oncogene C-myc, and tumour suppressor gene P53. [27–29]. Until now, studies have shown there are two main apoptotic pathways: the exogenous or death receptor pathway and the intrinsic or mitochondrial pathway [30]. Apoptosis may be directly or indirectly related to the occurrence and development of many diseases, such as lung cancer, COPD, asthma, atherosclerosis, diabetes, and autoimmune diseases [31, 32].

A study by Demedts et al. [33] indicated that apoptosis of lung structural cells may be an important upstream event in the pathogenesis of COPD. Both apoptotic alveolar epithelial and endothelial cells are increased in the lungs of patients with COPD. These pathological changes cannot be offset by the proliferation of structural cells, and this leads to the destruction of lung tissue and the development of emphysema.

**Fig. 1:**
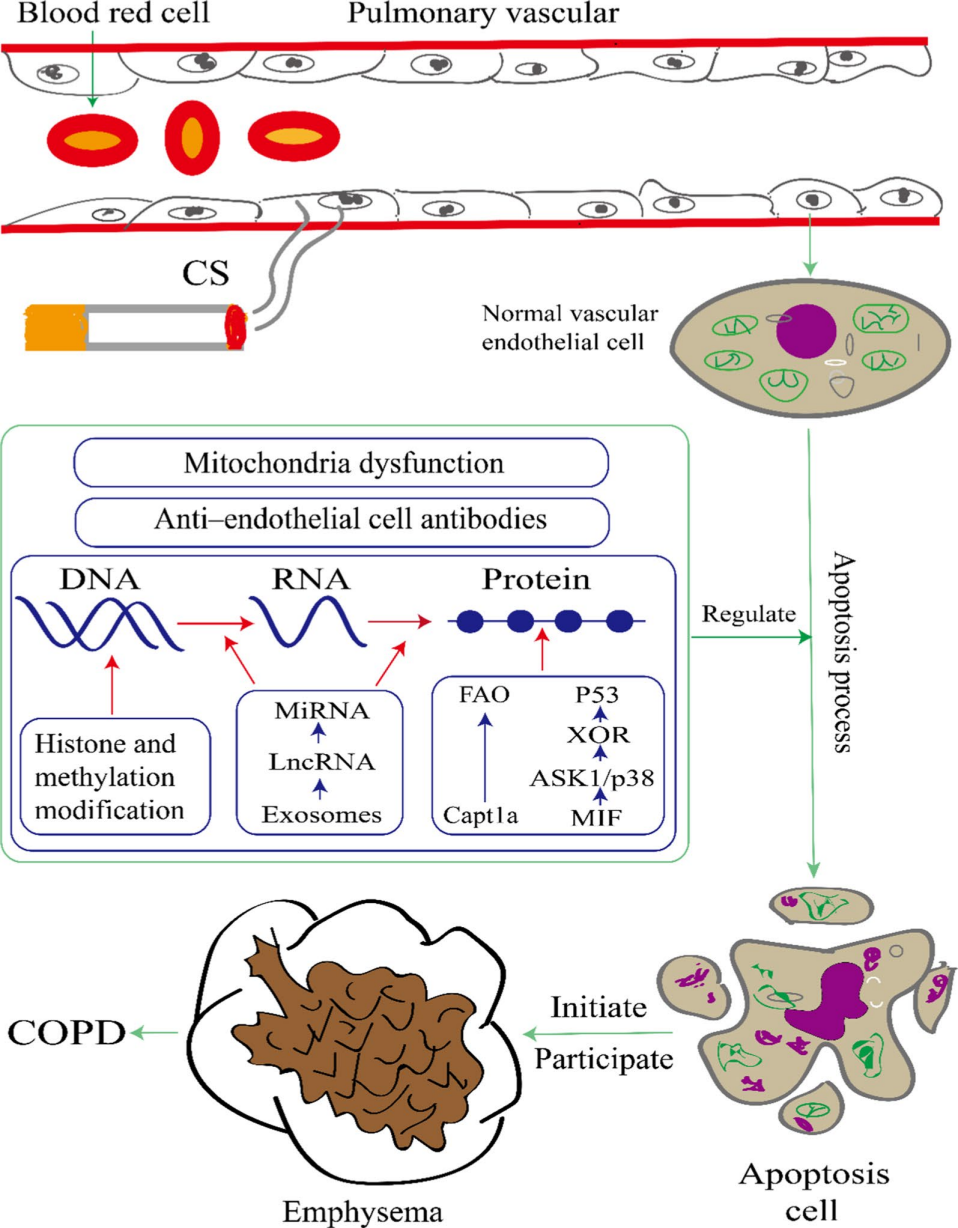
Epigenetic and other molecular biology mechanisms in cigarette smoke-induced pulmonary vascular endothelial cell apoptosis, which initiates and participates in the development of emphysema and COPD

Many studies have confirmed that pulmonary vascular endothelial cell apoptosis initiates and participates in the pathogenesis of COPD. Taraseviciene-Stewart et al. [34] induced the apoptosis of rat pulmonary vascular endothelial cells by intraperitoneal injection of cigarette smoke extract (CSE) in 2005 and successfully established a rat emphysema model. Successive studies have found that recombinant human tumour necrosis factors receptor: Fc fusion protein (rhTNFR: Fc) may interfere with tumour necrosis factor α (TNF-α) and reduce alveolar septal apoptosis in CS-induced rats [35]. In addition, vascular endothelial growth factor (VEGF) is one of the major regulators of endothelial cell survival and is believed to play a role in the pathogenesis of COPD [36]. Guan et al. [37] found that bone marrow mesenchymal stem cells could reduce pulmonary vascular endothelial cell apoptosis and promote cell survival by increasing VEGF expression in CS-induced rats. Moreover, Farkas et al. [38] found that after Smad3 knockout mice were exposed to CS, the expression of VEGF was reduced, which accelerated development of emphysema and COPD. Oral N-acetylcysteine may reduce emphysema and CS-induced alveolar septal cell apoptosis by partly increasing VEGF secretion and protein expression [39]. Chen et al. [40] induced the apoptosis of mouse pulmonary vascular endothelial cells with the intraperitoneal injection of CSE in 2009, also successfully establishing a mouse emphysema model, which provided a powerful guide for future research. With the deepening of research, more and more evidence has shown that epigenetic and other molecular biological mechanisms play an important role in regulating CSE/CS-induced apoptosis of pulmonary vascular endothelial cells. At the same time, a new chapter was opened for studying pulmonary vascular endothelial cell apoptosis initiating the development of COPD (Fig. 1).

#### Cigarette smoke and epigenetic mechanisms of pulmonary vascular endothelial cell apoptosis

Epigenetics refer to heritable changes of gene expression, without changing the nucleotide sequence of genes. More and more studies have shown that epigenetics is involved in the development of lung diseases. Epigenetic mechanisms, such as DNA methylation, RNA methylation, histone modification, exosomes (EXs), and non-coding RNA, with regulatory functions have been continuously revealed [41–43]. Studies have shown that long non-coding RNA (lncRNA), microRNA (miRNA) and DNA methylation, through various mechanisms to regulate the transcription of genes and proteins, and activate a series of molecular signal pathways to participate in the process of apoptosis [44–46](Table 1).

**Table 1:** Cigarette smoke and epigenetic mechanisms of pulmonary vascular endothelial cell apoptosis

Epigenetics	E group	C group	Detecting parameter	Detecting apoptosis cells	Comment	Reference
DNA methylation	Eleven COPD patients	Ten non-COPD patients	mtTFA, mtTFA promoter methylation	HPVECs	E group showing cell apoptosis increased, mtTFA mRNA and protein expression decreased. Methylation rate of the mtTFA promoter increased	Peng et al. [53]
	Ten BALB/c mice + CSE	Ten BALB/c mice + PBS	mtTFA, COXII, mtTFA promoter methylation	Mouse pulmonary vascular endothelial cells	E group showing cell apoptosis increased. mtTFA, COX II mRNA and protein expression decreased. Methylation rate of the mtTFA promoter increased	Zhang et al. [54]
	Ten COPD patients	Ten normal subjects	Notch1, ERK, mtTFA promoter methylation	HPMECs	E group showing cell apoptosis, ERK mRNA and protein expression increased. Notch1 mRNA and protein expression decreased. Methylation rate of the mtTFA promoter increased	Zong et al. [55]
	Ten BALB/c mice + CSE	Ten BALB/C mice + PBS	Bcl-2, Bax, Bcl-2 promoter methylation	Mice pulmonary vascular endothelial cells	E group showing cell apoptosis and Bax mRNA and protein expression increased. Bcl-2 mRNA and protein expression decreased. Methylation rate of the Bcl-2 promoter increased	Zeng et al. [56]
Histone methylation	HUVECs + CSE	HUVECs + PBS	PRMT6, H3R2me2a, H3K4me3	HUVECs	E group showing cell apoptosis and H3K4me3 protein expression increased. H3R2me2a protein expression decreased. PRMT6 mRNA and protein expression decreased	Kang et al. [72]
miRNA	HPMECs + CSE	HPMECs + PBS	miR-34a, Notch1	HPMECs	E group showing cell apoptosis and miR-34a expression increased, Notch1 mRNA and protein expression decreased	Long et al. [82]
	HPMECs + CSE	HPMECs + PBS	miR-206, Notch3, VEGFA	HPMECs	E group showing cell apoptosis and miR-206 expression increased. Notch3 and VEGFA mRNA and protein expression decreased	Sun et al. [83]
lncRNA	HPVECs + CSE	HPVECs + PBS	lncRNA MEG3, Bax, caspase-3, Bcl2	HPVECs	E group showing cell apoptosis and lncRNA MEG3 expression increased. Bax and caspase-3 expression increased. Bcl-2 increased	Bi et al. [97]
	HPMECs + CSE	HPMECs + PBS	lncRNA MIR155H, miRNA-218-5p, BRD4	HPMECs	E group showing cell apoptosis, lncRNA MIR155H and BRD4 expression increasedmiRNA-218-5p expression decreased	Song et al. [98]
Exosomes	RPMECs + CSE	RPMECs + PBS	Exosomes	RPMECs	Exosomes induced by 1% CSE significantly decreased the apoptosis rate of endothelial cells	Zhao et al. [105]

#### DNA methylation and pulmonary vascular endothelial cell apoptosis

DNA methylation is the most typical type of chromatin modification. It refers to changes of genetic expression, while without changes of DNA sequence. It is one of the common genetic modifications in epigenetics by adding a methyl group to the 5 'carbon position of the cytosine of the genomic CpG dinucleotide through the role of DNA methylation transferase [47, 48]. Research has shown that DNA methylation can regulate gene expression and participate in cell differentiation and apoptosis by changing DNA stability and structure [49]. Sundar et al. [50] isolated DNA from the lung tissue of eight non-smokers, eight current smokers, and eight patients with COPD and confirmed the presence of high DNA methylation in smokers and patients with COPD compared with non-smokers. Song et al. [51] isolated bronchial tissue from patients with and without COPD, isolated and cultured goblet cells and promoted their differentiation and found that SAM-pointed domain-containing ETS transcription factor (SPDEF) and forkhead box protein A2 (FOXA2) had abnormal DNA methylation during goblet cell differentiation. Zinellu et al. [52] studied the methylcytosine levels in the blood of forty-three patients with different degrees of COPD and forty-three control subjects. The results showed that DNA methylation was significantly increased in patients with COPD, especially patients with more severe COPD. These studies have shown that DNA methylation plays a key role in the pathogenesis of COPD.

Peng et al. [53] tested the rate of pulmonary vascular endothelial cell apoptosis in lung tissue of eleven patients with COPD and ten patients with non-COPD squamous cell lung cancer, measured the expression of mitochondrial transcription factor (mtTFA) mRNA and protein and methylation of the mtTFA promoter. The results showed that the patients with COPD had a higher cell apoptosis rate and lower mtTFA mRNA and protein expression compared with the non-COPD group, which has a negative correlation with pulmonary vascular endothelial cell apoptosis and smoke index. The methylation rate of the mtTFA promoter in the COPD group was significantly increased when compared with the non-COPD group. Zhang et al. [54] found that methylation of the mtTFA promoter and apoptosis rate of pulmonary vascular endothelial cells in the CSE-induced mice group were significantly increased. The mRNA and protein levels of both mtTFA and cytochrome c oxidase subunit II (COX II) were significantly decreased, but the group of mice treated with 5-aza-2′-deoxycytidine (AZA, a DNA methyltransferase inhibitor) had restoration of the above changes which suggesting that the removal of DNA methylation by AZA can protect against CSE-induced cell apoptosis. Zong et al. [55] tested the lung tissues of ten patients with COPD and ten normal subjects, respectively, and found that Notch1 was mainly expressed in endothelial cells, and was significantly decreased in the endothelial cells of patients with COPD. Furthermore, the results of in vitro cell experiments demonstrated that Notch1 overexpression reduces the CSE-induced apoptosis of human pulmonary microvascular endothelial cells (HPMECs), and CSE can significantly activate the extracellular signal-regulated kinase (ERK) signalling pathway. Treatment of CSE-induced HPMECs with ERK inhibitors can heavily reduce cell apoptosis and mtTFA methylation. Zeng et al. [56] studied the role and mechanisms of the Bcl protein family in the apoptosis of emphysema cells by intraperitoneal injection of CSE and AZA into mice, respectively, and found that the apoptosis index was higher than in the control group. The expression of Bcl-2 in CSE-induced mice decreased, but the level of Bcl-2 promoter methylation increased. However, AZA treatment promoted the Bcl-2 promoter demethylation, increased the expression of Bcl-2 and decreased the apoptosis index. These results indicated that the epigenetic mechanism of Bcl-2 promoter methylation is involved in CSE-induced emphysema and lung cell apoptosis.

#### Histone modification and pulmonary vascular endothelial cell apoptosis

Histone modification refers to histone acetylation, ubiquitination, phosphorylation, or methylation. Studies have shown that histone modification is involved in the regulation of gene expression at the epigenetic level and plays an important role in the development, ageing, differentiation, apoptosis, and tumour migration of tissues, organs and cells [57–62]. Sundar et al. [63] performed western blot analysis of targeted histones in lung tissue of CSE-induced mice and patients with COPD who continue to smoke. The results showed that the expression levels of several chromatin-modifying enzymes, including histone acetyltransferase, histone methyltransferase, histone domain proteins, and histone kinase were significantly increased. More studies have found that arginine methyltransferase-1 participates in the pathogenesis of epithelial tract injury in COPD by adding methyl to arginine residues in histones and non-histones to regulate protein modification at post-translational levels [64].

Chronic inflammation of the trachea and bronchi is one of the main characteristics of COPD [65]. Histone modification plays an important role in the chronic inflammation of COPD [66, 67]. Apoptosis of pulmonary vascular endothelial cells is one of the initiating events of COPD. Histone modification is also involved in smoking-induced emphysema and apoptosis [68–70]. He et al. [71] found that the expression of protein arginine methyltransferase 6 (PRMT6) and asymmetric di-methylation of histone H3 arginine 2 (H3R2me2a) were significantly decreased in the lung tissues of patients with COPD who continue to smoke and CSE-induced mice. However, H3R2me2a can prevent the tri-methylation of lysine 4 on histone H3 (H3K4me3) which is located at the transcription start site; the expression of H3K4me3 was significantly increased, and emphysema inflammation, apoptosis, and oxidative stress levels were more severe in CSE-induced mice. Further research found that apoptosis, emphysema inflammation, and oxidative stress were markedly reduced with overexpression of PRMT6. In other research, it was observed that the apoptosis of human umbilical vein endothelial cells (HUVECs) increased after CSE exposure and decreased PRMT6 expression. However, a decreased in CSE-induced apoptosis was observed after HUVECs were transfected with a plasmid expressing PRMT6. Notably, after CSE treatment, the expression of H3K4me3 protein significantly increased in HUVECs, while the expression of H3R2me2a protein decreased significantly in HUVECs. However, the above changes reversed after the transfection of cells with a plasmid expressing PRMT6, suggesting that PRMT6 mediated CSE-induced apoptosis through H3R2me2a in HUVECs [72].

#### miRNA and pulmonary vascular endothelial cell apoptosis

miRNA is a type of non-coding RNA with regulatory functions and a length of about 22–25 nucleotides. miRNA, which can regulate gene expression by incompletely or completely directly binding to mRNA 3′-untranslated region (UTR), also interacts with promoters, coding DNA sequence (CDS), and 5′-UTR to participate in gene regulation. It plays an important role in regulating gene expression, organism development, and apoptosis [73–77].

miRNA has been confirmed to be related to COPD and smoking. It plays an important role in the development of COPD [78, 79]. Conickx et al. [80] exposed mice to air and CS for twenty-four weeks and detected differential expression profiles of miRNAs in mice lung tissue and bronchoalveolar lavage fluid. The results showed that thirty-one miRNAs differentially expressed in lung tissue as well as seventy-eight miRNAs in bronchoalveolar lavage fluid in the CS exposed group compared with air exposure. Van Pottelberge et al. [81] also found that thirty-four miRNAs were differentially expressed in the sputum supernatants of patients who never smoked and current smokers. Compared with those who had never smoked and had no airflow limitation, the expression levels of eight miRNAs were significantly reduced in patients with COPD who continue to smoke.

In recent years, the role of miRNA in the smoking-induced apoptosis of pulmonary vascular endothelial cells and its related mechanisms also have been studied. Research by Long et al. [82] showed that CSE can induce apoptosis of HPMECs with miR-34a significantly upregulated. The miRNA target gene library was further predicted through a biological information database and Notch1 was determined to be the target of miRNA-34a. At the same time, it was confirmed that miR-34a regulates gene expression at post-transcriptional levels by targeting Notch1 mRNA 3′-UTR after luciferase gene determination. Furthermore, studies have confirmed that the expression level of Notch1 in CSE-induced HPMECs is markedly decreased. In vitro cell experiments also confirmed that miR-34a mimic and Notch1 gene plasmids were transfected into HPMECs exposed to CSE. Overexpression of miR-34a can significantly increase the apoptosis rate of HPMECs. However, the overexpression of Notch1 has a protective effect on the apoptosis of HPMECs caused by the increased miR-34a and reduced the apoptosis rate. In other research, CSE-induced HPMECs significantly upregulate miR-206 levels. However, the cell apoptosis rate decreased after the miR-206 gene was knocked out. miR-206 participates in the regulation of gene expression by targeting Notch3 and vascular endothelial growth factor A (VEGFA) mRNA 3′-UTR after prediction by the bioinformatics gene database. Then, miR-206 mimic, Notch3 vector plasmid and VEGFA vector plasmid were transfected into CSE-induced HPMECs, respectively. The results showed that overexpression of miR-206 can lead to increased apoptosis of HPMECs. However, the overexpression of Notch3 and VEGFA can significantly reduce apoptosis [83].

#### lncRNA and pulmonary vascular endothelial cell apoptosis

lncRNA is non-protein-coding RNA with a length longer than 200 nucleotides. lncRNA can regulate target gene expression through different mechanisms to participate in cell biological processes, from chromatin regulation to protein degradation [84, 85], such as interacting with miRNAs via ceRNA or directly target miRNAs, by encoding proteins or polypeptides and by means of intercellular communication through EXs [86–89].

lncRNA has also has been confirmed to be related to COPD and smoking [90, 91]. Qian et al. [92] identified the differentially expressed lncRNA in smoking and non-smoking patients with COPD through RNA sequencing and bioinformatics analysis and found that ninety-six lncRNA were identified in non-smoking patients, while forty-four lncRNA were identified in smoking patients with COPD. Further research into the lncRNA-miRNA-mRNA interaction network found that let-7c-ADRB1-HLA-DQB1-AS1 interactions may play a key role in the pathogenesis in smoking COPD patients. Let-7c was downregulated in smokers with COPD associated with forced expiratory volume in 1 s. Adrenoceptor beta 1 (ADRB1) is a subtype of the adrenergic receptors which are involved with the management of COPD exacerbations and is upregulated in smoking COPD patients [93]. HLA-DQB1-AS1 was a co-expressed lncRNA of ADRB1 and was upregulated in smoking COPD patients. Otherwise, there were one hundred and nine lncRNA were also found to be differentially expressed compared to control groups in lung tissue of CS-induced COPD mice models [94]. Studies have shown that lncRNA TUG1 can promote airway remodelling by inhibiting the miR‐145‐5p and DUSP6 axis in a CS-induced COPD mice model [95]. A study by Zhang et al. [96] found that the lncRNA NEAT1 can inhibit the apoptosis of t-BHP-treated HUVECs by activating the miR-181d-5p and cyclin-dependent kinase inhibitor 3 (CDKN3) axis. Bi et al. [97] study indicated that co-cultured human pulmonary vascular endothelial cells (HPVECs) with different concentrations of CSE (0%, 0.1%, 1% and 10%) significantly promoted cell apoptosis, increased caspase-3 activity, upregulated Bax expression, decreased Bcl-2 expression, and increased expression of the lncRNA MEG3. After the transfection of lncRNA MEG3 with a plasmid, the expression of lncRNA MEG3 was increased, and cell apoptosis further increased. However, knockdown of lncRNA MEG3 showed the opposite effect, decreased cell apoptosis, decreased caspase activity, decreased Bax expression, and upregulated Bcl-2 expression. Also, Song et al. [98] found that the expression of the lncRNA MIR155HG was increased, while miRNA-218-5p was decreased in CSE-induced HPMECs. Subsequently, it was found that miRNA-218-5p was a direct target of MIR155HG. This result was also confirmed in the rescue experiment, as a miRNA-218-5p inhibitor reduced the inhibition effect of MIR155HG on CSE-induced HPMECs. Further studies showed that miRNA-218-5p directly targeted bromodomain containing 4 (BRD4), and overexpression of miRNA-218-5p reversed cell apoptosis by regulating BRD4. In conclusion, MIR155HG participates in the apoptosis of CSE-induced HPMECs by regulating the miRNA-218-5p and BRD4 axis.

#### Exosomes and pulmonary vascular endothelial cell apoptosis

EXs are extracellular vesicles (EVs) with a size of approximately 30–150 nm that produce inward budding originating from the endosomal membrane of the cell upon activation or during apoptosis [99]. It has been demonstrated that EXs play a key role in intercellular communication by carrying biomolecules, including proteins, DNA, miRNA and lncRNA, involved in cell communication, migration, angiogenesis, and proliferation [100].

Some studies have demonstrated that CS can promote the release of EXs in lung tissue cells. Benedikter et al. [101] revealed that CSE exposure could boost the number of EXs secreted by bronchial epithelial cells. In addition, exposure to tobacco smoke extract (TSE) exposure can cause human macrophages to release EVs (including exosomes and ectosomes), which contribute to the release of matrix metalloproteinase 14 (MMP14) and may contribute to emphysema [102, 103]. Studies have found that MMP14 activity and protein was increased in the airway epithelium of tobacco smoke-exposed mice and decreased MMP14 activity and protein could diminish the mucin 5AC, oligomeric mucus/gel-forming (MUC5AC) transcripts that played significant roles in the development of COPD [104]. A study by Zhao et al. [105] showed that CS-induced epithelial cell-derived EXs decreased the apoptosis of rat pulmonary microvascular endothelial cells, but the underlying mechanisms remain unclear and need further research.

### Cigarette smoke and other molecular biology mechanisms of pulmonary vascular endothelial cell apoptosis

There are still related studies exploring the mechanisms of pulmonary vascular endothelial cell apoptosis in other molecular biology caused by smoking [106]. The metabolism of the three major nutrients of protein, fat and glucose are the basis of the body's life activities and the basic component of cells. It maintains the stability of cellular structure and participates in the life activities of cells [107]. Studies have shown that glucose production, clearance, oxidation, and glycolysis rates are increased in patients with COPD compared to healthy subjects [108]. In addition, CS exposure has been shown to reduce glycolysis in type II cells [109]. Similarly, lipid metabolism disorders also exist during acute exacerbations of COPD. Glycerophospholipid and sphingomyelin metabolism are associated with airflow obstruction, decreased lung function, and exacerbation of COPD [110, 111]. Decreased levels of lipoproteins and amino acids were also observed in the serum and urine of patient with COPD [112]. In another study of pulmonary microvascular endothelial cells in CSE-induced mice and patient with COPD, the authors found that the carnitine palmitoyl transferase 1a (Cpt1a) in cells was significantly reduced. In turn, the oxidative ability of fatty acids (FAO) and mitochondrial respiration were decreased, but the apoptosis was increased. Further studies also verified similar results. CSE-induced apoptosis was further increased when pulmonary microvascular endothelial cells were treated with Cpt1 inhibitor or transfected with Cpt1a siRNA. Treatment with L-carnitine increased the amount of FAO and reduced cell apoptosis by increasing Cpt1a expression [113]. A study by Wang et al. [114] found that the mitochondrial aberrations, fission, oxidative stress, and cell apoptosis were increased, while mitochondrial respiration and fusion were decreased in CSE-induced rat lung microvascular endothelial cells (RLMVECs). However, barrier dysfunction and apoptosis decreased in CS-induced RLMVECs after inhibition of mitochondrial fission and anti-oxidant intervention of mitochondria.

There were studies found that in the systemic, CS-induced endothelial dysfunction through the following aspects: firstly, directed toxic effects of CS on endothelial cells; then, promoted the production of auto-antibodies in endothelial cells; next, CS-induced inflammation of vascular; in addition, increased oxidative stress levels with reduced activation of the anti-oxidant pathways in endothelial cells; finally, CS-induced increased mediators with vasoconstrictor, pro-inflammatory, and remodelling activities and increased endoplasmic reticular stress and the unfolded protein response in endothelial cells [115]. A study by Taraseviciene-Stewart et al. [116] found that intraperitoneal injection of endothelial cells into rats could lead to the generation of anti-endothelial cell antibodies, which promoted endothelial cell apoptosis and caused emphysema. However, concomitant injection with the toll-like receptor 4 (TLR4) ligand lipid A into rats could decrease endothelial cell apoptosis and reduce the incidence of emphysema. It was implied that CS induction might lead to the generation of anti-endothelial cell antibodies, which promoted vascular endothelial cell apoptosis and caused emphysema. However, it needed further research. In addition, a study by Romundstad et al. [117] found that renal dysfunction was linked to CS-induced lung injury, with an association between emphysema severity and the estimated glomerular filtration rate. In addition, patients with COPD who were shown to have more glomerulosclerosis and greater renal arterial and arteriolar sclerosis were linked to vascular endothelial cell injury and apoptosis [118]. It might be that the oxidative stress level was increased which further activated the advanced glycation end product (AGE) and receptor for advanced glycation end products (RAGE) in CS-induced endothelial cells and circulating CS directed toxicity on endothelial cells. Also, the production of anti-endothelial antibodies against endothelial cells. So, it was worthy to further study the connection between lung and kidney endothelial cell injury and apoptosis in patients with COPD with CS [119].

Cell activity is a complex process which is regulated by multiple genes and protein molecules. Cyclooxygenase-2 is a rate-limiting enzyme in the metabolic pathway of cells and can convert arachidonic acid into prostaglandins. Studies have demonstrated that CSE can affect the expression of cyclooxygenase-2 in HPVECs, subsequently affecting the production of prostaglandins. It is worth noting that prostaglandins can inhibit the CSE-induced apoptosis of vascular endothelial cells [120]. P53 is a tumour suppressor gene that encodes the p53 protein involved in the process of cell apoptosis. Macrophage migration inhibitory factor (MIF) is an anti-apoptotic cytokine produced by HPVECs. The expression of MIF was decreased, while P53 was increased when pulmonary vascular endothelial cells were exposed to CSE. However, MIF is a negative regulator of p53 expression and can protect the CSE-induced apoptosis of pulmonary vascular endothelial cells by combating p53-mediated caspase-dependent apoptosis pathways [121]. Another study also found that xanthine oxidoreductase (XOR) is an upstream effector of p53. XOR activity was significantly increased in the lung tissues of CS-induced mice, promoted the production of ROS, and involved in CS-induced pulmonary vascular endothelial cells apoptosis through the p53-mediated caspase-dependent apoptosis pathway [122]. Also, XOR activity was significantly increased in the bronchoalveolar lavage fluid of patients with COPD [123, 124]. Interestingly, Fallica et al. [125] found that MIF, a pleiotropic cytokine, both reduced in mice with CS-induced emphysema and patients with COPD. Further studies have found that MIF, as a determinant factor of ROS production after vascular endothelial cells were exposed to CS, affected the apoptosis signal-regulating kinase 1 (ASK1) P38 kinase cascade, regulated the activity of XOR enzymes produced by ROS and antagonized ASK1-p38-dependent pulmonary vascular endothelial cell apoptosis. In general, MIF reduces the CS-induced apoptosis of pulmonary vascular endothelial cells by inhibiting the signal transduction of the ASK1-P38-XOR pathway (Table 2).

**Table 2:** Cigarette smoke and other molecular biology mechanisms of pulmonary vascular endothelial cell apoptosis

E group	C group	Detecting parameter	Detecting apoptosis cells	Comment	Reference
Mouse PMVECs + CSE	Mouse PMVECs + PBS	Mitochondrial respiration, FAO, oxidative phosphorylation, Cpt1a, ceramide synthesis	PMVECs	E group showing cell apoptosis increased. Oxidative phosphorylation, FAO and Cpt1a decreased. Mitochondrial respiration decreased. Ceramide synthesis increased	Gong et al. [113]
COPD patients PMVECs	Healthy subjects PMVECs	Mitochondrial respiration, FAO, oxidative phosphorylation, Cpt1a, ceramide synthesis	Lung tissue cells	E group showing cell apoptosis increased. Oxidative phosphorylation, FAO and Cpt1a decreased. Mitochondrial respiration decreased. Ceramide synthesis increased	
Rat PMVECs + CSE	Rat PMVECs + PBS	Mitochondrial morphology, oxidative stress, respiration; fission and fusion	PMVECs	E group showing cell apoptosis, mitochondrial aberrations, fission and mitochondrial oxidative stress increased. Mitochondrial respiration and fusion was decreased	Wang et al. [114]
ECV304 + CSE	ECV304 + PBS	COX II	ECV304	E group showing cell apoptosis and COX II protein expression increased	Shi et al. [122]
Six smokers with COPD	Seven non-smokers without COPD and seven smokers without COPD	COX II	Vascular endothelial cells in lung tissues	E group showing AI of medium-sized vessels increased. COX II protein expression increased	
HPAECs + CSE	HPAECs + PBS	P53, MIF	HPAECs	E group showing cell apoptosis increased. P53 and MIF mRNA and protein expression increased	Damico et al. [123]
C57BL/6 male mice + CS	C57BL/6 male mice + AIR	XOR protein and activity, alveolar diameter	Non	E group showing alveolar diameter enlargement. XOR protein expression and XOR activity increased	Kim et al. [124]
HLMVECs + CSE	HLMVECs + PBS	XOR protein and activity, P53	HLMVECs	E group showing cell apoptosis increased XOR protein expression and XOR activity increased. P53 mRNA and protein expression increased	
Mif−/− C57BL/6 Mice + CS	Mif + / + C57BL/6 Mice + CS	XOR activity, P38 protein, ASK1, caspase 3/7 protein and activity	Human and rat lung microvascular Endothelial cells	E group showing cell apoptosis increased. P38 protein increased. ASK1 and caspase3/7 protein and activity increased	Fallica et al. [127]

In addition, CS-induce pulmonary vascular endothelial cell apoptosis could promote the secretion of transforming growth factor-beta 1(TGF-β1) [126, 127]. TGF-β1 is a multi-functional cytokine that regulates angiogenesis, and fibroblasts/myofibroblasts [128]. Moreover, the TGF-β1/Smad2.3 signalling pathway is strongly implicated in endothelial to mesenchymal transition (EndMT) which plays a key role in the pathogenesis of COPD [129–131]. At the same time, accumulated research on EndMT showed that endothelial dysfunction contributes to the pathogenesis of pulmonary hypertension [132] and pulmonary vascular endothelial cell apoptosis can promote the development of pulmonary hypertension, which is a common complication of COPD and is closely related to COPD progression [133]. Studies have shown that the reticular basement membrane (Rbm) had markedly increased splitting and hypervascularity, while the lamina propria (LP) was hypovascular in COPD. Inhaled corticosteroid (ICS) therapy increased the density of vessels and brought back it to normal levels in the LP, but there was no influence on the Rbm hypervascular, which may suggest that ICS therapy reduces vessel destruction rather than promotes the growth of new vessels. This might be related to vascular endothelial cell apoptosis, but the specific mechanisms need further investigation [134, 135].

## Conclusions

**Fig. 2:**
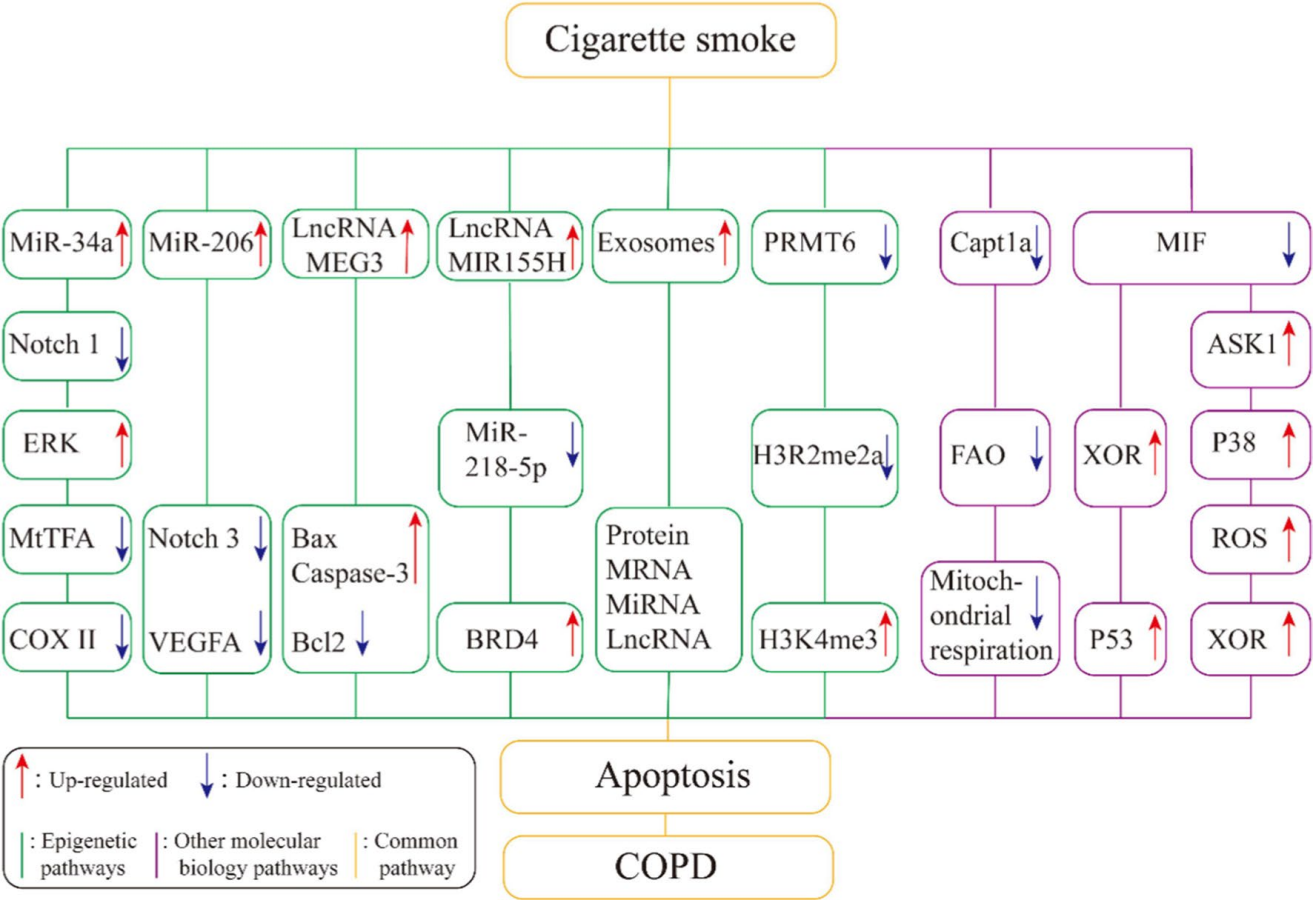
CS-induced apoptosis of pulmonary vascular endothelial cells, epigenetic and other molecular biological mechanisms of regulatory pathways

CS is one of the main causes of COPD, and it induces pulmonary vascular endothelial cell apoptosis initiates and participates the pathogenesis of COPD. However, the mechanisms of CS-induced apoptosis have not been fully elucidated. Epigenetics has been a hot topic in recent years. Histone modification, miRNA, lncRNA, DNA methylation, RNA methylation and other regulatory effects also exist in the CS-induced pulmonary vascular endothelial cell apoptosis. However, its potential regulatory mechanisms need to be further studied. The development of molecular biology technology provides the possibility to discover and study the underlying mechanisms of COPD. Elucidating the mechanisms of CS-induced pulmonary vascular endothelial cell apoptosis will help to explore new strategies in the diagnosis, treatment, and prevention of COPD (Fig. 2).

## Data Availability

All publications discussed in the manuscript are available from the corresponding author on request.

## Abbreviations

AGE: Advanced glycation end products
AZA: 5-Aza-2′-deoxycytidine
ADRB1: Adrenoceptor beta 1
BRD4: Bromodomain containing 4
C group: Control group
COPD: Chronic obstructive pulmonary disease
CS: Cigarette smoke
CSE: Cigarette smoke extract
CDS: Coding DNA sequence
Cpt1a: Carnitine palmitoyl transferase 1a
COX II: Cytochrome c oxidase subunit II
CDKN3: Cyclin-dependent kinase inhibitor 3
ERK: Extracellular signal-regulated kinase
EXs: Exosomes
EVs: Extracellular vesicles
E group: Experiment group
EndMT: Endothelial-to-mesenchymal
FOXA2: Fork head box protein A2
FAO: Fatty acids
H3K4me3: Tri-methylation of H3K4
H3R2me2a: Asymmetric di-methylation of histone H3 arginine 2
HPMECs: Human pulmonary microvascular endothelial cells
HUVECs: Human umbilical vein endothelial cells
HLMVECs: Human lung microvascular endothelial cells
HPVECs: Human pulmonary vascular endothelial cell
HPAECs: Human pulmonary artery endothelial cells
ICS: Inhaled corticosteroid. lncRNA: long non-coding RNA
LP: Lamina propria
MIF: Macrophage migration inhibitory factor
MtTFA: Mitochondrial transcription factor
MMP14: Matrix metalloproteinases 14
MUC5AC: Mucin 5AC, oligomeric mucus/gel-forming
PRMT6: Protein arginine methyltransferase 6
PMVECs: Pulmonary microvascular endothelial cells
PBS: Phosphate buffered saline
RPMECs: Rat pulmonary microvascular endothelial cells
Rbm: Reticular basement membrane
ROS: Reactive oxygen species
RLMVECs: Rat lung microvascular endothelial cells
RAGE: Receptor for advanced glycation end products
SPDEF: SAM-pointed domain-containing Ets-like factor
TNF-α: Tumor necrosis factor-α
rhTNFR: Fc: Recombinant human tumour necrosis factors receptor Fc fusion protein
TLR4: Toll-like receptor 4
TGF-β1: Transition transforming growth factor-beta 1
TSE: Tobacco smoke extract
VEGFA: Vascular endothelial growth factor A
VEGF: Vascular endothelial growth factor. XOR: Xanthine oxidoreductase
3′- UTR: 3′-Untranslated regions (UTR)

## References

[1] Zinellu E, Zinellu A, Fois AG, Fois SS, Piras B, Carru C (2020). Reliability and usefulness of different biomarkers of oxidative stress in chronic obstructive pulmonary disease. Oxid Med Cell Longev.

[2] Smith MC, Wrobel JP (2014). Epidemiology and clinical impact of major comorbidities in patients with COPD. Int J Chron Obstruct Pulmon Dis.

[3] Miravitlles M, Ribera A (2017). Understanding the impact of symptoms on the burden of COPD. Respir Res.

[4] Yan F, Gao H, Zhao H, Bhatia M, Zeng Y (2018). Roles of airway smooth muscle dysfunction in chronic obstructive pulmonary disease. J Transl Med.

[5] GBD 2015 Disease and Injury Incidence and Prevalence Collaborators (2016). Global, regional, and national incidence, prevalence, and years lived with disability for 310 diseases and injuries, 1990–2015: a systematic analysis for the Global Burden of Disease Study 2015. Lancet.

[6] GBD 2015 Chronic Respiratory Disease Collaborators (2017). Global, regional, and national deaths, prevalence, disability-adjusted life years, and years lived with disability for chronic obstructive pulmonary disease and asthma, 1990–2015: a systematic analysis for the Global Burden of Disease Study 2015. Lancet Respir Med..

[7] Adeloye D, Chua S, Lee C, Basquill C, Papana A, Theodoratou E (2015). Global and regional estimates of COPD prevalence: systematic review and meta-analysis. J Glob Health.

[8] GBD Chronic Respiratory Disease Collaborators (2020). Prevalence and attributable health burden of chronic respiratory diseases, 1990–2017: a systematic analysis for the Global Burden of Disease Study 2017. Lancet Respir Med.

[9] Li X, Cao X, Guo M, Xie M, Liu X (2020). Trends and risk factors of mortality and disability adjusted life years for chronic respiratory diseases from 1990 to 2017: systematic analysis for the Global Burden of Disease Study 2017. BMJ.

[10] Zhu B, Wang Y, Ming J, Chen W, Zhang L (2018). Disease burden of COPD in China: a systematic review. Int J Chron Obstruct Pulmon Dis.

[11] Hou W, Hu S, Li C, Ma H, Wang Q, Meng G, Guo T, Zhang J (2019). Cigarette smoke induced lung barrier dysfunction, EMT, and tissue remodeling: a possible link between COPD and lung cancer. Biomed Res Int.

[12] Zong D, Liu X, Li J, Chen P (2019). The role of cigarette smoke-induced epigenetic alterations in inflammation. Epigenetics Chromatin.

[13] Baraldo S, Turato G, Saetta M (2012). Pathophysiology of the small airways in chronic obstructive pulmonary disease. Respiration.

[14] Leopold PL, O’Mahony MJ, Lian XJ, Tilley AE, Harvey BG, Crystal RG (2009). Smoking is associated with shortened airway cilia. PLoS ONE.

[15] Maestrelli P, Saetta M, Mapp CE, Fabbri LM (2001). Remodeling in response to infection and injury. Airway inflammation and hypersecretion of mucus in smoking subjects with chronic obstructive pulmonary disease. Am J Respir Crit Care Med..

[16] Van Eeden SF, Hogg JC (2019). Immune-modulation in chronic obstructive pulmonary disease: current concepts and future strategies. Respiration..

[17] Domej W, Oettl K, Renner W (2014). Oxidative stress and free radicals in COPD–implications and relevance for treatment. Int J Chron Obstruct Pulmon Dis.

[18] Yoshida T, Tuder RM (2007). Pathobiology of cigarette smoke-induced chronic obstructive pulmonary disease. Physiol Rev.

[19] Zinellu E, Zinellu A, Fois AG, Carru C, Pirina P (2016). Circulating biomarkers of oxidative stress in chronic obstructive pulmonary disease: a systematic review. Respir Res.

[20] Wang C, Xu J, Yang L, Xu Y, Zhang X, Bai C, Kang J (2018). Prevalence and risk factors of chronic obstructive pulmonary disease in China (the China Pulmonary Health [CPH] study): a national cross-sectional study. Lancet.

[21] Yin P, Jiang CQ, Cheng KK, Lam TH, Lam KH, Miller MR (2007). Passive smoking exposure and risk of COPD among adults in China: the Guangzhou Biobank Cohort Study. Lancet.

[22] Mannino DM, Buist AS (2007). Global burden of COPD: risk factors, prevalence, and future trends. Lancet.

[23] Hagstad S, Bjerg A, Ekerljung L, Backman H, Lindberg A, Rönmark E, Lundbäck B (2014). Passive smoking exposure is associated with increased risk of COPD in never smokers. Chest.

[24] Barnes PJ, Burney PG, Silverman EK, Celli BR, Vestbo J, Wedzicha JA (2015). Chronic obstructive pulmonary disease. Nat Rev Dis Primers.

[25] Zeng H, Kong X, Zhang H, Chen Y, Cai S, Luo H (2020). Inhibiting DNA methylation alleviates cigarette smoke extract-induced dysregulation of Bcl-2 and endothelial apoptosis. Tob Induc Dis.

[26] Elmore S (2007). Apoptosis: a review of programmed cell death. Toxicol Pathol Toxicol Pathol.

[27] Schuler M, Green DR (2001). Mechanisms of p53-dependent apoptosis. Biochem Soc Trans.

[28] Oda E, Ohki R, Murasawa H, Nemoto J, Shibue T, Yamashita T (2000). Noxa, a BH3-only member of the Bcl-2 family and candidate mediator of p53-induced apoptosis. Science.

[29] Green DR, Llambi F (2015). Cell death signaling. Cold Spring Harb Perspect Biol.

[30] Igney FH, Krammer PH (2002). Death and anti-death: tumour resistance to apoptosis. Nat Rev Cancer.

[31] Braithwaite AT, Marriott HM, Lawrie A (2018). Divergent roles for TRAIL in lung diseases. Front Med (Lausanne).

[32] Turkmen K (2017). Inflammation, oxidative stress, apoptosis, and autophagy in diabetes mellitus and diabetic kidney disease: the Four Horsemen of the Apocalypse. Int Urol Nephrol.

[33] Demedts IK, Demoor T, Bracke KR (2006). Role of apoptosis in the pathogenesis of COPD and pulmonary emphysema. Respir Res.

[34] Taraseviciene-Stewart L, Scerbavicius R, Choe KH, Moore M, Sullivan A (2005). An animal model of autoimmune emphysema. Am J Respir Crit Care Med.

[35] Zhang C, Cai S, Chen P, Chen JB, Wu J, Wu SJ, Zhou R (2008). Inhibition of tumor necrosis factor-alpha reduces alveolar septal cell apoptosis in passive smoking rats. Chin Med J.

[36] Farid M, Kanaji N, Nakanishi M, Gunji Y, Michalski J, Iwasawa S (2013). Smad3 mediates cigarette smoke extract (CSE) induction of VEGF release by human fetal lung fibroblasts. Toxicol Lett.

[37] Guan XJ, Song L, Han FF, Cui ZL, Chen X, Guo XJ (2013). Mesenchymal stem cells protect cigarette smoke-damaged lung and pulmonary function partly via VEGF-VEGF receptors. J Cell Biochem.

[38] Farkas L, Farkas D, Warburton D, Gauldie J, Shi W, Stampfli MR (2011). Cigarette smoke exposure aggravates air space enlargement and alveolar cell apoptosis in Smad3 knockout mice. Am J Physiol Lung Cell Mol Physiol.

[39] Cai S, Chen P, Zhang C, Chen JB, Wu J (2009). Oral N-acetylcysteine attenuates pulmonary emphysema and alveolar septal cell apoptosis in smoking-induced COPD in rats. Respirology.

[40] Chen Y, Hanaoka M, Chen P (2009). Protective effect of beraprost sodium, a stablel prostacyclin analogue, in the development of cigarette smoke extract-induced emphysema. Am J Physiol Lung Cell Mol Physiol.

[41] Duru S (2016). Epigenetic and current treatment approaches in chronic obstructive pulmonary disease. Tuberk Toraks.

[42] Alashkar Alhamwe B, Alhamdan F, Ruhl A, Potaczek DP, Renz H (2020). The role of epigenetics in allergy and asthma development. Curr Opin Allergy Clin Immunol.

[43] Mehta A, Dobersch S, Romero-Olmedo AJ, Barreto G (2015). Epigenetics in lung cancer diagnosis and therapy. Cancer Metastasis Rev.

[44] Liu D, Zhang H, Cong J (2020). H3K27 acetylation-induced lncRNA EIF3J-AS1 improved proliferation and impeded apoptosis of colorectal cancer through miR-3163/YAP1 axis. J Cell Biochem.

[45] Shen Q, Zheng J, Wang X, Hu W, Jiang Y, Jiang Y (2020). lncRNA SNHG5 regulates cell apoptosis and inflammation by miR-132/PTEN axis in COPD. Biomed Pharmacother.

[46] Li F, Zhang C, Zhang G (2019). M6A RNA methylation controls proliferation of human glioma cells by influencing cell apoptosis. Cytogenet Genome Res.

[47] Moore LD, Le T, Fan G, Moore LD (2013). DNA methylation and its basic function. Neuropsychopharmacology..

[48] Youn HD (2017). Methylation and demethylation of DNA and histones in chromatin: the most complicated epigenetic marker. Exp Mol Med.

[49] Pan Y, Liu G, Zhou F, Su B, Li Y (2018). DNA methylation profiles in cancer diagnosis and therapeutics. Clin Exp Med.

[50] Sundar IK, Yin Q, Baier BS, Yan L, Mazur W, Li D, Susiarjo M, Rahman I (2017). DNA methylation profiling in peripheral lung tissues of smokers and patients with COPD. Clin Epigenetics.

[51] Song J, Heijink IH, Kistemaker LEM (2017). Aberrant DNA methylation and expression of SPDEF and FOXA2 in airway epithelium of patients with COPD. Clin Epigenetics.

[52] Zinellu A, Sotgiu E, Fois AG, Zinellu E, Sotgia S, Ena S (2017). Blood global DNA methylation is decreased in non-severe chronic obstructive pulmonary disease (COPD) patients. Pulm Pharmacol Ther.

[53] Peng H, Yang M, Chen ZY, Chen P, Guan CX, Xiang XD (2013). Expression and methylation of mitochondrial transcription factor a in chronic obstructive pulmonary disease patients with lung cancer. PLoS ONE.

[54] Zhang H, Chen P, Zeng H, Zhang Y, Peng H, Chen Y, He Z (2013). Protective effect of demethylation treatment on cigarette smoke extract-induced mouse emphysema model. J Pharmacol Sci.

[55] Zong D, Li J, Cai S, He S, Liu Q, Jiang J (2018). Notch1 regulates endothelial apoptosis via the ERK pathway in chronic obstructive pulmonary disease. Am J Physiol Cell Physiol.

[56] Zeng H, Shi Z, Kong X, Chen Y, Zhang H, Peng H, Luo H, Chen P (2016). Involvement of B-cell CLL/lymphoma 2 promoter methylation in cigarette smoke extract-induced emphysema. Exp Biol Med (Maywood).

[57] Peleg S, Feller C, Ladurner AG, Imhof A (2016). The metabolic impact on histone acetylation and transcription in ageing. Trends Biochem Sci.

[58] Huang Y, Zou Y, Lin L (2017). Effect of BIX-01294 on proliferation, apoptosis, and histone methylation of acute T lymphoblastic leukemia cells. Leuk Res.

[59] Brehove M, Wang T, North J, Luo Y, Dreher SJ (2015). Histone core phosphorylation regulates DNA accessibility. J Biol Chem.

[60] Tushir-Singh J, Bhatnagar S (2017). In vitro assay to study histone ubiquitination during transcriptional regulation. Methods Mol Biol.

[61] Yao H, Rahman I (2012). Role of histone deacetylase 2 in epigenetics and cellular senescence: implications in lung inflammaging and COPD. Am J Physiol Lung Cell Mol Physiol..

[62] Kouzarides T (2007). Chromatin modifications and their function. Cell.

[63] Sundar IK, Rahman I (2016). Gene expression profiling of epigenetic chromatin modification enzymes and histone marks by cigarette smoke: implications for COPD and lung cancer. Am J Physiol Lung Cell Mol Physiol.

[64] Sarker RSJ, Conlon TM, Morrone C, Srivastava B, Konyalilar N, Verleden SE (2019). CARM1 regulates senescence during airway epithelial cell injury in COPD pathogenesis. Am J Physiol Lung Cell Mol Physiol..

[65] Barnes PJ (2017). Cellular and molecular mechanisms of asthma and COPD. Clin Sci (Lond).

[66] Chen TT, Wu SM, Ho SC, Chuang HC, Liu CY, Chan YF (2017). SUV39H1 reduction is implicated in abnormal inflammation in COPD. Sci Rep.

[67] Kaur G, Bagam P, Pinkston R, Singh DP, Batra S (2018). Cigarette smoke-induced inflammation: NLRP10-mediated mechanisms. Toxicology.

[68] Kim SY, Lee JH, Huh JW, Ro JY, Oh YM, Lee SD (2011). Cigarette smoke induces Akt protein degradation by the ubiquitin-proteasome system. J Biol Chem.

[69] Andresen E, Gunther G, Bullwinkel J, Lange C, Heine H (2011). Increased expression of beta-defensin 1(DEFB1) in chronic obstructive pulmonary disease. PLoS ONE.

[70] Banerjee A, Koziol-White C, Panettieri R (2012). P38 MAPK inhibitors, IKK2 inhibitors, and TNFα inhibitors in COPD. Curr Opin Pharmacol.

[71] He X, Li T, Kang N, Zeng H, Ren S, Zong D (2017). The protective effect of PRMT6 overexpression on cigarette smoke extract-induced murine emphysema model. Int J Chron Obstruct Pulmon Dis.

[72] Kang N, Chen P, Chen Y, Zeng H, He X, Zhu Y (2015). PRMT6 mediates CSE induced inflammation and apoptosis. Int Immunopharmacol.

[73] O'Brien J, Hayder H, Zayed Y, Peng C (2018). Overview of microRNA Biogenesis, Mechanisms of Actions, and Circulation. Front Endocrinol (Lausanne).

[74] Yan S, Shi J, Sun D, Lu L (2020). Current insight into the roles of microRNA in vitiligo. Mol Biol Rep.

[75] Qadir MI, Faheem A (2017). miRNA: a diagnostic and therapeutic tool for pancreatic cancer. Crit Rev Eukaryot Gene Expr.

[76] Jiang J, Xia Y, Liang Y, Yang M, Zeng W, Zeng X (2018). miR-190a-5p participates in the regulation of hypoxia-induced pulmonary hypertension by targeting KLF15 and can serve as a biomarker of diagnosis and prognosis in chronic obstructive pulmonary disease complicated with pulmonary hypertension. Int J Chron Obstruct Pulmon Dis.

[77] Atherton LJ, Jorquera PA, Bakre AA, Tripp RA (2019). Determining Immune and miRNA Biomarkers Related to Respiratory Syncytial Virus (RSV) Vaccine Types. Front Immunol.

[78] Gu W, Yuan Y, Yang H, Wu H, Wang L, Tang Z, Li Q (2018). Role of miR-195 in cigarette smoke-induced chronic obstructive pulmonary disease. Int Immunopharmacol.

[79] Zeng Z, He S, Lu J, Liu C, Li H, Xu C, Xie L, Sun S (2018). microRNA-21 aggravates chronic obstructive pulmonary disease by promoting autophagy. Exp Lung Res.

[80] Conickx G, Avila Cobos F, van den Berge M (2017). microRNA profiling in lung tissue and bronchoalveolar lavage of cigarette smoke-exposed mice and in COPD patients: a translational approach. Sci Rep.

[81] Van Pottelberge GR, Mestdagh P, Bracke KR, Faiz A, Timens W, Hiemstra PS (2011). microRNA expression in induced sputum of smokers and patients with chronic obstructive pulmonary disease. Am J Respir Crit Care Med.

[82] Long YJ, Liu XP, Chen SS, Zong DD, Chen Y, Chen P (2018). miR-34a is involved in CSE-induced apoptosis of human pulmonary microvascular endothelial cells by targeting Notch-1 receptor protein. Respir Res.

[83] Sun Y, An N, Li J, Xia J, Tian Y, Zhao P (2019). miRNA-206 regulates human pulmonary microvascular endothelial cell apoptosis via targeting in chronic obstructive pulmonary disease. J Cell Biochem.

[84] Li J, Li Z, Zheng W, Li X, Wang Z, Cui Y, Jiang X (2017). lncRNA-ATB: An indispensable cancer-related long noncoding RNA. Cell Prolif.

[85] Jathar S, Kumar V, Srivastava J, Tripathi V (2017). Technological developments in lncRNA biology. Adv Exp Med Biol.

[86] Guo W, Yu Q, Zhang M, Li F, Liu Y, Jiang W, Jiang H, Li H (2019). Long intergenic non-protein coding RNA 511 promotes the progression of osteosarcoma cells through sponging microRNA 618 to upregulate the expression of maelstrom. Aging (Albany NY).

[87] Song B, Ye L, Wu S, Jing Z (2020). Long non-coding RNA MEG3 regulates CSE-induced apoptosis and inflammation via regulating miR-218 in 16HBE cell. Biochem Biophys Res Commun.

[88] Tang J, Yu B, Li Y, Zhang W, Alvarez AA, Hu B (2019). TGF-β-activated lncRNA LINC00115 is a critical regulator of glioma stem-like cell tumorigenicity. EMBO Rep.

[89] Chen L, Yang W, Guo Y, Che W, Zheng P, Zeng J, Tong W (2017). Exosomal lncRNA GAS5 regulates the apoptosis of macrophages and vascular endothelial cells in atherosclerosis. PLoS ONE.

[90] De Smet EG, Mestdagh P, Vandesompele J, Brusselle GG, Bracke KR (2015). Non-coding RNAs in the pathogenesis of COPD. Thorax.

[91] Zheng M, Hong W, Gao M, Yi E, Zhang J, Hao B (2019). Long noncoding RNA COPDA1 promotes airway smooth muscle cell proliferation in chronic obstructive pulmonary disease. Am J Respir Cell Mol Biol.

[92] Qian Y, Mao ZD, Shi YJ, Liu ZG, Cao Q, Zhang Q (2018). Comprehensive analysis of miRNA-mRNA-lncRNA networks in non-smoking and smoking patients with chronic obstructive pulmonary disease. Cell Physiol Biochem.

[93] Antonelli-Incalzi R, Pedone C (2007). Respiratory effects of beta-adrenergic receptor blockers. Curr Med Chem.

[94] Zhang H, Sun D, Li D, Zheng Z, Xu J, Liang X (2019). Long non-coding RNA expression patterns in lung tissues of chronic cigarette smoke induced COPD mouse model. Sci Rep.

[95] Gu W, Yuan Y, Wang L (2019). Long non-coding RNA TUG1 promotes airway remodeling by suppressing the miR-145-5p/DUSP6 axis in cigarette smoke-induced COPD. J Cell Mol Med.

[96] Zhang M, Wang X, Yao J, Qiu Z (2019). Long non-coding RNA NEAT1 inhibits oxidative stress-induced vascular endothelial cell injury by activating the miR-181d-5p/CDKN3 axis. Artif Cells Nanomed Biotechnol.

[97] Bi H, Wang G, Li Z, Zhou L, Zhang M, Ye J, Wang Z (2020). Long noncoding RNA (lncRNA) Maternally Expressed Gene 3 (MEG3) participates in chronic obstructive pulmonary disease through regulating human pulmonary microvascular endothelial cell apoptosis. Med Sci Monit.

[98] Song J, Wang Q, Zong L (2020). LncRNA MIR155HG contributes to smoke-related chronic obstructive pulmonary disease by targeting miR-128–5p/BRD4 axis. Biosci Rep..

[99] Poulet C, Njock MS, Moermans C, Louis E, Louis R, Malaise M (2020). Int J Mol Sci.

[100] Kourembanas S (2015). Exosomes: vehicles of intercellular signaling, biomarkers, and vectors of cell therapy. Annu Rev Physiol.

[101] Benedikter BJ, Volgers C, Eijck PH, Wouters EFM, Savelkoul PHM (2017). Cigarette smoke extract induced exosome release is mediated by depletion of exofacial thiols and can be inhibited by thiol-antioxidants. Free Radic Biol Med.

[102] Li M, Yu D, Williams KJ, Liu ML (2010). Tobacco smoke induces the generation of procoagulant microvesicles from human monocytes/macrophages. Arterioscler Thromb Vasc Biol.

[103] O'Farrell HE, Yang IA (2019). Extracellular vesicles in chronic obstructive pulmonary disease (COPD). J Thorac Dis.

[104] Deshmukh HS, McLachlan A, Atkinson JJ, Hardie WD, Korfhagen TR, Dietsch M (2009). Matrix metalloproteinase-14 mediates a phenotypic shift in the airways to increase mucin production. Am J Respir Crit Care Med.

[105] Zhao Y, Yang L, Zhou A (2019). Cigarette smoke-induced epithelial cell-derived exosomes regulate the apoptosis of endothelial cells. Chest.

[106] Nana-Sinkam SP, Lee JD, Sotto-Santiago S, Stearman RS (2007). Prostacyclin prevents pulmonary endothelial cell apoptosis induced by cigarette smoke. Am J Respir Crit Care Med.

[107] Goñi FM (2014). The basic structure and dynamics of cell membranes: an update of the Singer-Nicolson model. Biochim Biophys Acta.

[108] Li J, Lu Y, Li N, Li P, Wang Z, Ting W (2020). Chemerin: a potential regulator of inflammation and metabolism for chronic obstructive pulmonary disease and pulmonary rehabilitation. Biomed Res Int.

[109] Agarwal AR, Yin F, Cadenas E (2014). Short-term cigarette smoke exposure leads to metabolic alterations in lung alveolar cells. Am J Respir Cell Mol Biol.

[110] Jiang Z, Knudsen NH, Wang G, Qiu W, Naing ZZC, Bai Y (2017). Genetic control of fatty acid β-oxidation in chronic obstructive pulmonary disease. Am J Respir Cell Mol Biol.

[111] Cruickshank-Quinn CI, Jacobson S, Hughes G, Powell RL, Petrache I (2018). Metabolomics and transcriptomics pathway approach reveals outcome-specific perturbations in COPD. Sci Rep.

[112] Wang L, Tang Y, Liu S, Mao S, Ling Y, Liu D (2013). Metabonomic profiling of serum and urine by (1)H NMR-based spectroscopy discriminates patients with chronic obstructive pulmonary disease and healthy individuals. PLoS ONE.

[113] Gong J, Zhao H, Liu T, Li L, Cheng E, Zhi S (2019). Cigarette smoke reduces fatty acid catabolism, leading to apoptosis in lung endothelial cells: implication for pathogenesis of COPD. Front Pharmacol.

[114] Wang Z, White A, Wang X, Ko J, Choudhary G, Lange T (2020). Mitochondrial fission mediated cigarette smoke-induced pulmonary endothelial injury. Am J Respir Cell Mol Biol.

[115] Polverino F, Celli BR, Owen CA (2018). COPD as an endothelial disorder: endothelial injury linking lesions in the lungs and other organs? (2017 Grover Conference Series). Pulm Circ.

[116] Taraseviciene-Stewart L, Scerbavicius R, Choe KH, Moore M, Sullivan A, Nicolls MR (2005). An animal model of autoimmune emphysema. Am J Respir Crit Care Med.

[117] Romundstad S, Naustdal T, Romundstad PR, Sorger H, Langhammer A (2014). COPD and microalbuminuria: a 12-year follow-up study. Eur Respir J.

[118] Polverino F, Laucho-Contreras ME, Petersen H (2017). A pilot study linking endothelial injury in lungs and kidneys in chronic obstructive pulmonary disease. Am J Respir Crit Care Med.

[119] Voelkel NF (2018). Cigarette Smoke Is an Endothelial Cell Toxin. Am J Respir Crit Care Med.

[120] Lunghi B, De Cunto G, Cavarra E, Fineschi S, Bartalesi B, Lungarella G, Lucattelli M (2015). Smoking p66Shc knocked out mice develop respiratory bronchiolitis with fibrosis but not emphysema. PLoS ONE.

[121] Chen H, Liao K, Cui-Zhao L, Qiang-Wen F, Feng-Zeng X, Ping-Wu F, Liang-Guo S, Juan-Chen Y (2015). Cigarette smoke extract induces apoptosis of rat alveolar type II cells via the PLTP/TGF-β1/Smad2 pathway. Int Immunopharmacol.

[122] Shi Z, Chen Y, Pei Y, Long Y, Liu C, Cao J, Chen P (2017). The role of cyclooxygenase-2 in the protection against apoptosis in vascular endothelial cells induced by cigarette smoking. J Thorac Dis.

[123] Damico R, Simms T, Kim BS, Tekeste Z, Amankwan H (2011). P53 mediates cigarette smoke-induced apoptosis of pulmonary endothelial cells: inhibitory effects of macrophage migration inhibitor factor. Am J Respir Cell Mol Biol.

[124] Kim BS, Serebreni L, Hamdan O, Wang L, Parniani A, Sussan T (2013). Xanthine oxidoreductase is a critical mediator of cigarette smoke-induced endothelial cell DNA damage and apoptosis. Free Radic Biol Med.

[125] Pinamonti S, Muzzoli M, Chicca MC, Papi A, Ravenna F, Fabbri LM (1996). Xanthine oxidase activity in bronchoalveolar lavage fluid from patients with chronic obstructive pulmonary disease. Free Radic Biol Med.

[126] Pinamonti S, Leis M, Barbieri A, Leoni D, Muzzol M, Sostero S (1998). Detection of xanthine oxidase activity products by EPR and HPLC in bronchoalveolar lavage fluid from patients with chronic obstructive pulmonary disease. Free Radic Biol Med..

[127] Fallica J, Varela L, Johnston L, Kim B, Serebreni L, Wang L (2016). Macrophage migration inhibitory factor: a novel inhibitor of apoptosis signal-regulating kinase 1–p38-xanthine oxidoreductase-dependent cigarette smoke-induced apoptosis. Am J Respir Cell Mol Biol.

[128] Kranenburg AR, de Boer WI, Alagappan VK, Sterk PJ, Sharma HS (2005). Enhanced bronchial expression of vascular endothelial growth factor and receptors (Flk-1 and Flt-1) in patients with chronic obstructive pulmonary disease. Thorax.

[129] Sohal SS (2017). Epithelial and endothelial cell plasticity in chronic obstructive pulmonary disease (COPD). Respir Investig..

[130] Mahmood MQ, Reid D, Ward C, Muller HK, Knight DA, Sohal SS, Walters EH (2017). Transforming growth factor (TGF) beta1 and Smad signalling pathways: a likely key to EMT-associated COPD pathogenesis. Respirology (Carlton, Vic).

[131] Sohal SS (2016). Endothelial to mesenchymal transition (EndMT): an active process in Chronic Obstructive Pulmonary Disease (COPD)?. Respir Res.

[132] Gaikwad AV, Eapen MS, McAlinden KD, Chia C, Larby J, Myers S (2020). Endothelial to mesenchymal transition (EndMT) and vascular remodeling in pulmonary hypertension and idiopathic pulmonary fibrosis. Expert Rev Respir Med.

[133] Xue C, Sowden M, Berk BC (2017). Extracellular cyclophilin A, especially acetylated, causes pulmonary hypertension by stimulating endothelial apoptosis, redox stress, and inflammation. Arterioscler Thromb Vasc Biol.

[134] Soltani A, Reid DW, Sohal SS, Wood-Baker R, Weston S, Muller HK (2010). Basement membrane and vascular remodelling in smokers and chronic obstructive pulmonary disease: a cross-sectional study. Respir Res.

[135] Soltani A, Walters EH, Reid DW, Shukla SD, Nowrin K, Ward C (2016). Inhaled corticosteroid normalizes some but not all airway vascular remodeling in COPD. Int J Chron Obstruct Pulmon Dis.

